# Association of Polymorphisms in HLA Antigen Presentation-Related Genes with the Outcomes of HCV Infection

**DOI:** 10.1371/journal.pone.0123513

**Published:** 2015-04-13

**Authors:** Peng Huang, Yuanyuan Zhang, Xiaomei Lu, Yin Xu, Jie Wang, Yun Zhang, Rongbin Yu, Jing Su

**Affiliations:** 1 Department of Epidemiology and Biostatistics, School of Public Health, Nanjing Medical University, Nanjing, China; 2 Department of General Practice, Kangda College, Nanjing Medical University, Nanjing, China; 3 Department of Epidemiology, Medical Institute of Nanjing Army, Nanjing, China; Centers for Disease Control and Prevention, UNITED STATES

## Abstract

Antigen-presentation genes play a vital role in the pathogenesis of HCV infection. However, the relationship of variants of these genes with spontaneous outcomes of HCV infection has not been fully investigated. To explore novel loci in the Chinese population, 34 tagging-SNPs in 9 candidate genes were genotyped for their associations with the outcomes of HCV infection. The distributions of different genotypes and haplotypes were compared among 773 HCV-negative controls, 246 subjects with HCV natural clearance, and 218 HCV persistent carriers recruited from hemodialysis patients and intravenous drug users. Our study implicated that *TAP2*, *HLA-DOA*, *HLA-DOB*, and *tapasin* loci were novel candidate regions for susceptibility to HCV infection and viral clearance in the Chinese population. Logistic regression analyses showed that *TAP2* rs1800454 A (OR = 1.48, *P *= 0.002) and *HLA-DOB* rs2071469 G (OR = 1.23, *P *= 0.048) were significantly associated with increased susceptibility to establishment of HCV infection. However, high-risk behavior exposure and age were stronger predictors of HCV infection. Mutation of *tapasin* rs9277972 T (OR = 1.57, *P *=0.043) increased the risk of HCV chronicity, and *HLA-DOA* rs3128935 C (OR = 0.62, *P *= 0.019) increased the chance of viral resolution. With regards to the effect of rs3128925, interactions were found with high-risk behavior (*P* = 0.013) and age (*P* = 0.035). The risk effect of rs3128925 T for persistent HCV infection was higher in injecting drug users (vs. dialysis patients) and in subjects ≥ 40 years old (vs. < 40 years old).

## Introduction

Hepatitis C virus (HCV) infection is one of the most common chronic blood-borne infections in the world. It is estimated that up to 170 million people are infected with HCV, with 29 million in China [1]. HCV is transmitted by percutaneous exposure to contaminated blood, perinatal exposure from a mother to her infant, unprotected sexual intercourse, and drug use. Approximately 30% of individuals spontaneously clear acute HCV infection, while the remaining become chronically infected. About 30% of chronic patients will develop chronic liver disease, including cirrhosis and hepatocellular carcinoma (HCC) [2]. Many factors are associated with the different clinical outcomes (HCV clearance or persistence), and studies have demonstrated that a strong host immune response against HCV favors viral clearance [3]. Thus, variations in the genes involved in the immune response may contribute to the outcome of infectious diseases, including HCV infection.

The primary function of human leukocyte antigen (HLA) complex is to provide protection against pathogens. One important step of the host immune response is the presentation of HCV antigens in the context of HLA molecules [4, 5]. The classical regions of HLA are the most extensively studied regions with regard to disease association; however, some non-classical genes have not been sufficiently investigated, such as the family of antigen presentation-related genes mainly located in the HLA class II *DQ-DP* interval on the short arm of human chromosome 6. There are eight genes classified in this family in the *DQ-DP* interval, including *TAP1*, *TAP2*, *LMP2*, *LMP7*, *HLA-DMA*, *HLA-DMB*, *HLA-DOA*, and *HLA-DOB* [6, 7]. *TAP1* and *TAP2* encode the two subunits of TAP (transporter associated with antigen processing). TAP works with its binding protein, TAPBP, which is encoded by *tapasin* located near the centromere of chromosome 6; thus, the *tapasin* gene is also categorized in the family of antigen presentation-related genes [8]. *LMP2* and *LMP7* encode LMP (large molecular weight proteasome). The TAP and LMP transport antigenic peptides from the cytosol into the endoplasmic reticulum in an ATP-dependent manner [9]. These peptides are then assembled with HLA class I heavy chain and β2-microglobulin. The *HLA-DMA* and *HLA*-*DMB* genes encode a DM molecule similar to HLA class II molecules, which are involved in antigen processing. DM is required for the efficient release of class II-associated Ii peptide (CLIP) and the assembly of antigenic peptides with the HLA class II molecules for transport to the cell surface [10]. DO is also a two subunit molecule composed of DMA and DMB, and negatively regulates the mechanisms of HLA-DM [5, 11].

Several studies have evaluated the relationships between antigen presentation-related genes and the response to interferon (IFN) treatment in chronic HCV patients and many human autoimmune diseases, such as Grave’s Disease (GD), type 1 diabetes mellitus (T1D), and rheumatoid arthritis (RA) [12–17]. The aim of this study is to investigate whether variants of antigen presentation-related genes might be associated with the outcomes of HCV infection. Therefore, we genotyped 34 tagging-SNPs in 9 candidate genes (*HLA-DMA*, *HLA-DMB*, *HLA-DOA*, *HLA-DOB*, *TAP1*, *TAP2*, *LMP2*, *LMP7*, *tapasin*) in a high risk population of HCV infection in China.

## Materials and Methods

### Participants

The participants of injecting drug users (IDUs) were recruited from the Nanjing compulsory detoxification center during May and Dec 2006 (479 drug users), and hemodialysis patients were recruited from nine hospital hemodialysis centers in southern China during Oct 2008 and Jan 2010 (758 hemodialysis patients). The study protocol was approved by the institutional review committee of Nanjing Medical University for ethical issues. Signed informed consent was obtained from each participant beforehand. Interviews for demographic characteristics and risk factors were conducted from May 2006 to Jan 2010. A blood sample (~10 mL) was then collected for serological tests and host DNA genotyping. The subjects included in the final cohort for this study were negative of the following conditions: hepatitis B surface antigen (HBsAg), other types of liver diseases, alcoholic diseases, metabolic liver diseases, and previous interferon and/or ribavirin therapy.

### Laboratory analysis and genotyping of SNPs

The subjects’ serological status of HBsAg and HCV antibody (anti-HCV) was detected by enzyme-linked immunosorbent assay (Beijing Wantai Biological Pharmacy Engineering Co., Ltd., Beijing, China) following the manufacturer’s instructions. Blood biochemical tests were undertaken by Roche MODULE P800 Automatic Biochemical Analyzer (Roche Co., Ltd., Shanghai, China). Total serum RNA was extracted using Trizol LS Reagent (TaKaRa Biotechnology Co., Ltd., Dalian, China), and HCV RNA was detected by RT-PCR using RT-PCR kit (TaKaRa Biotechnology Co., Ltd., Dalian, China). HCV genotyping was performed as previously described [14, 15]. The Murex HCV Serotyping 1–6 Assay ELISA Kit (Abbott, Wiesbaden, Germany) was used to test the type-specific antibodies to HCV genotypes 1 to 6 in sera with non-detectable HCV RNA [18]. The subjects were classified into the following groups: 1. the uninfected controls who were anti-HCV free; 2. the infected cases who were anti-HCV positive. The infected cases were further divided into two groups: chronic cases (HCV RNA positive) and spontaneous resolvers (HCV RNA negative).

DNA extraction was performed by protease K digestion and phenol-chloroform purification as described previously [19]. Single-nucleotide polymorphisms (SNPs) in 9 candidate genes (*HLA-DMA*, *HLA-DMB*, *HLA-DOA*, *HLA-DOB*, *TAP1*, *TAP2*, *LMP2*, *LMP7*, *tapasin*) were selected from NCBI dbSNP database (http://www.ncbi.nlm.nih.gov/SNP) and the public HapMap SNP database (http://www.hapmap.org). All the SNPs were filtered with the following criteria: (1) MAF ≥ 0.05 in Chinese Han population; (2) Hardy-Weinberg equilibrium test *P* value ≥ 0.05. Overall, 34 tagging-SNPs were chosen for further genotyping. Genotyping was performed by the TaqMan allelic discrimination assay on ABI PRISM 7900HT Sequence Detection system (Applied Biosystems, San Diego, CA, USA). The information of SNPs and probes is shown in S1 Table and S2 Table. Two blank controls and five repeated samples were assigned into each 384-well format as quality control measures, and a 100% concordant was achieved. The success rates of SNPs genotyping were above 95%, and the observed genotype frequencies in subjects were in Hardy-Weinberg equilibrium.

### Statistical analysis

General demographic characteristics were compared by the Student’s *t*-test, One-Way ANOVA, and the chi-square (*χ* ^2^) test. The associations of SNPs with HCV infection outcomes were estimated by the odds ratios (ORs) and 95% confidence intervals (CIs) using both univariate and multivariate logistic regression analyses. Adjustment for age, gender, high-risk population, and/or viral genotype was conducted during regression analyses. The trend analysis was assessed with Cochran-Armitage trend test. All of the statistical analyses were carried out by R software (version 2.14.0; The R Foundation for Statistical Computing), and *P* < 0.05 in a two-sided test was considered as statistically significant. Meanwhile, to avoid spurious associations with false positive outcomes, the Benjamini-Hochberg false discovery rate (BH-FDR) correction was performed. FDR < 0.3 were accepted as statistically significant. In order to evaluate the predicted importance of significant SNPs and other risk factors, the random forest model was used during analyses. Receiver operating characteristic (ROC) curves were drawn to demonstrate the prediction values of the risk factors. The plot of the variables’ importance and the area under the curve (AUC) indicated the results [20, 21].

## Results

All enrolled participants were divided into three groups including 773 anti-HCV negative controls, 246 spontaneous clearance cases (anti-HCV positive and HCV RNA negative), and 218 persistent HCV cases (both anti-HCV and HCV RNA positive). Some selected demographic characteristics were compared among the groups and the results were shown in Table 1. Distribution of gender was the same among the three groups (*P* = 0.052). However, significant differences were observed for age, high-risk population, and viral genotype (*P* < 0.001). HCV prevalence was higher in IDUs than in hemodialysis patients (61.80% vs. 22.16%), indicating intravenous drug use increased chance for infection. Over 70% of the cases were infected with HCV 1b type (solely or mixed with other viral types). The level of aspartate aminotransferase (AST) and alanine aminotransferase (ALT) indicated that the liver function of infected subjects was worse compared with that of non-infected subjects.

**Table 1:** Demographic and selected variables in subjects with different HCV infection outcomes.

Variables	Uninfected	Resolver	Chronic	*P* Value
	(n = 773) N(%)	(n = 246) N(%)	(n = 218) N(%)	
Age, year (mean ± SD)	46.94 ± 15.28	39.10 ±12.17	40.47 ± 12.33	1.70×10^–13^
Gender				5.20×10^–2^
Male	485 (62.7)	156 (63.4)	156 (71.6)	
Female	288 (37.3)	90 (36.6)	62 (28.4)	
High-risk population				2.21×10^–13^
Hemodialysis patient	590 (76.3)	92 (37.4)	76 (34.8)	
Injecting drug user	183 (23.7)	154 (62.6)	142 (65.2)	
Viral genotype				1.56×10^–4^
Genotype-1b	—	174 (71.0)	186 (86.1)	
Non-1b^*^	—	71 (29.0)	30 (13.9)	
AST (U/L)				1.37×10^–12^
< 40	724 (93.7)	214 (87.0)	165 (75.7)	
≥ 40	49 (6.3)	32 (13.0)	53 (24.3)	
ALT (U/L)				2.20×10^–16^
< 40	727 (94.0)	199 (80.9)	149 (68.3)	
≥ 40	46 (6.0)	47 (19.1)	69 (31.7)	

Abbreviation: SD, standard deviation.

^*^Non-1b means viral strains other than 1b including genotype 1a, 2, and 3 (either solely or mixed infection).

### Effect of polymorphisms of Antigen-presentation genes on HCV susceptibility and clearance

Distributions of 34 tagging-SNPs from 9 genes were investigated for association with the outcomes of HCV infection. Dominant, recessive, and additive genetic models were used in analysis of each SNP. Significance in any model was considered as a possible association of these SNPs with HCV infection. To control the type I error during multiple comparisons, FDR were also calculated. The comparison results of genetic models and FDR were provided in S2 Table. After adjusting for age, gender, high-risk population, and/or viral genotype, logistic regression analyses showed that *HLA-DOA*, *HLA*-*DOB*, *TAP2*, and *tapasin* were associated with different outcomes of HCV infection. For *HLA-DOA* rs3128935 and *tapasin* rs9277972, FDR was 0.26 under additive model.

The full infection process of HCV involves the following steps of acquisition, colonization, penetration, spread, damage, and resolution [22]. Acquisition of HCV may result in nothing more than a brief encounter. After establishing a replicative niche, the virus may invade the host, penetrating the anatomical barrier and causing tissue damage. While an uninfected subject may simply acquire the virus without viral invasion, an infected subject will undergo the full viral invasion process. The host immune system will mount a response to this invasion, so the presence of anti-HCV is used as the biomarker to distinguish between uninfected and infected subjects. Furthermore, the ability to clear the virus determines whether an infected subject is a resolver or a chronic case.

The allelic frequencies of antigen presentation-related genes were first compared among the uninfected controls and infected individuals. The *HLA-DOB* rs2071469 G and *TAP2* rs1800454 A mutants significantly increased the risk of being invaded by HCV (Table 2A). Multivariate stepwise regression analyses were performed with age, sex, high-risk population, and these two SNPs. The results showed that *HLA-DOB* rs2071469 (*P* = 0.048), *TAP2* rs1800454 (*P* = 0.002), and high risk population (*P* = 3.20×10^–6^) were independent factors of HCV infection (Table 3A). Therefore, these two SNPs were included in further Cochran-Armitage’s trend test. When combining the unfavorable genotypes of rs1800454-AG/AA and rs2071469-AG/GG, the infection risk did not increase (*P* = 0.648) (Table 4A).

**Table 2:** Association of selected SNPs with HCV infection outcomes.

**a) SNPs associated with anti-HCV condition**				
**Genotype**	**Uninfected N(%)**	**Infected N(%)**	**OR (95% CI)** ^**a**^	***P* value** ^**a**^
rs1800454				
GG	594 (76.8)	322 (69.4)	1.00	
AG	158 (20.4)	125 (26.9)	1.60 (1.19–2.16)	2.00×10^–3^
AA	21 (2.8)	17 (3.7)	1.48 (0.72–3.03)	2.83×10^–1^
Dominant			1.59 (1.19–2.11)	1.00×10^–3^
Recessive			1.32 (0.65–2.69)	4.42×10^–1^
Additive			1.44 (1.13–1.82)	3.00×10^–3^
rs2071469				
AA	429 (55.5)	300 (64.6)	1.00	
AG	228 (29.5)	129 (27.8)	1.39 (1.03–1.88)	3.10×10^–2^
GG	116 (15.0)	35 (7.6)	1.20 (0.76–1.87)	4.33×10^–1^
Dominant			1.34 (1.01–1.79)	4.00×10^–2^
Recessive			1.03 (0.67–1.57)	9.02×10^–1^
Additive			1.17 (0.96–1.43)	1.23×10^–1^
**b) SNPs associated with HCV chronicity**				
**Genotype**	**Resolver N(%)**	**Chronic N(%)**	**OR (95% CI)** ^**b**^	***P* value** ^**b**^
rs9277972				
AA	206 (83.7)	164 (75.2)	1.00	
AT	38 (15.4)	50 (22.9)	1.75 (1.08–2.85)	2.40×10^–2^
TT	2 (0.9)	4 (1.9)	2.63 (0.46–15.14)	2.78×10^–1^
Dominant			1.80 (1.12–2.88)	1.60×10^–2^
Recessive			2.34 (0.41–13.42)	3.39×10^–1^
Additive			1.72 (1.11–2.66)	1.50×10^–2^
rs3128935				
TT	175 (71.1)	174 (79.8)	1.00	
CT	63 (25.6)	43 (19.7)	0.69 (0.44–1.10)	1.18×10^–1^
CC	8 (3.3)	1 (0.5)	0.11 (0.01–0.89)	3.80×10^–2^
Dominant			0.62 (0.40–0.97)	3.60×10^–2^
Recessive			0.12 (0.02–0.96)	4.60×10^–2^
Additive			0.59 (0.40–0.89)	1.20×10^–2^

^a^ Logistic regression analyses adjusted for age, gender, and high-risk population.

^b^ Logistic regression analyses adjusted for age, gender, high-risk population, and viral genotype.

**Table 3:** Multivariate stepwise regression analysis for independent factors of HCV infection outcomes.

**a) anti-HCV condition**				
**Variables**	**Coef.**	**SE**	**OR (95% CI)** ^**a**^	***P* value** ^**a**^
rs1800454	0.392	0.184	1.48 (1.16–1.89)	2.00×10^–3^
rs2071469	0.205	0.127	1.23 (1.00–1.50)	4.80×10^–2^
High-risk population	1.870	0.939	6.49 (4.89–8.62)	3.20×10^–6^
**b) HCV chronicity**				
**Variables**	**Coef.**	**SE**	**OR (95% CI)** ^**b**^	***P* value** ^**b**^
rs9277972	0.454	0.353	1.57(1.02–2.44)	4.30×10^–2^
rs3128935	-0.475	0.126	0.62(0.42–0.93)	1.90×10^–2^
Viral genotype	0.921	0.607	2.51(1.56–4.03)	2.70×10^–4^

Abbreviation: Coef, coefficient of variation; SE, standard error.

^a^ Logistic regression analyses adjusted for high-risk population.

^b^ Logistic regression analyses adjusted for viral genotype.

**Table 4:** Cumulative effects of selected SNPs on HCV infection outcomes.

**a) Combined unfavorable genotypes (rs18004545-AG/AA and rs2071469-AG/GG) and anti-HCV**				
**Variables**	**Uninfected N(%)**	**Infected N(%)**	**OR (95% CI)** ^**a**^	***P* value** ^**a**^
0	318 (41.1)	205 (44.2)	1.00	-
1	387 (50.1)	212 (45.7)	1.41 (1.06–1.87)	1.70×10^–2^
2	68 (8.8)	47 (10.1)	2.43 (1.53–3.87)	1.69×10^–4^
Trend			*P* ^c^ = 0.648	
0	318 (41.1)	205 (44.2)	1.00	-
1–2	455 (58.9)	259 (55.8)	1,53 (1.16–2.01)	2.03×10^–2^
**b) Combined unfavorable genotypes (rs9277975-AT/TT and rs3128935-TT) and HCV chronicity**				
**Variables**	**Resolver N(%)**	**Chronic N(%)**	**OR (95% CI)** ^**b**^	***P* value** ^**b**^
0	64 (26.0)	32 (14.7)	1.00	-
1	149 (60.6)	144 (66.1)	2.00 (1.21–3.30)	6.04×10^–3^
2	33 (13.4)	42 (19.2)	2.69 (1.41–5.13)	4.39×10^–3^
Trend			*P* ^c^ = 0.002	
0	64 (26.0)	32 (14.7)	1.00	-
1–2	182 (74.0)	186 (85.3)	2.13 (1.30–3.47)	3.41×10^–3^

^a^ Logistic regression analyses adjusted for age, gender, and high-risk population.

^b^ Logistic regression analyses adjusted for age, gender, high-risk population, and viral genotype.

^c^ *P* value of Cochran-Armitage’s trend test.

After invasion of the virus, the ability to clear the virus also determines the different clinical outcomes. The ability of viral clearance was then compared between the spontaneous infection subjects and the persistent infection subjects. The *tapasin* rs9277972 T mutant significantly increased the risk of HCV persistence, while the *HLA-DOA* rs3128935 C mutant increased the percentage of HCV clearance (Table 2B). The multivariate stepwise regression analyses showed that rs9277972 (*P* = 0.043), rs3128935 (*P* = 0.019), and viral genotype (*P* = 2.70×10^–4^) were independent factors of chronicity (Table 3B). Therefore, the combined effects of rs9277972-AT/TT and rs3128935-TT were analyzed in Cochran-Armitage’s trend test. There was an increased risk of persistent infection with the more unfavorable genotypes (*P* = 0.002). Subjects carrying two unfavorable genotypes had a 169% increase in risk of chronic HCV infection (OR = 2.69, 95% CI = 1.41–5.13), as shown in Table 4B.

### Multivariate analysis of factors associated with the outcomes of HCV

We used decision tree ensembles in the form of a random forest classifier to quantify the relative predictive power of the SNPs, age, gender, high-risk population, and viral genotype. As a result of this multivariate analysis, the predictive power of one variable is expressed as the Gini. High-risk population and age were the top two strong predictors to estimate the chance of being infected by HCV. The rs2071469 and rs1800454 followed at position 3 and 4. When combining the two SNPs into one classifier, the area under the ROC curve was 0.570, which was rather low. Better test performance was obtained by adding high-risk exposure or age into the model. The set of high-risk exposure, rs2071469 and rs1800454 yielded an AUC of 0.727, and the set of high-risk exposure, age, rs2071469 and rs1800454 yielded the best AUC of 0.730. These results indicated that in addition to carrying unfavorable SNPs, unsafe behaviors and old age increase the risk of HCV infection. To predict the ability of viral clearance, rs3128935, rs9277972, and viral genotype were the top three strong predictors. The AUC was 0.563 for a set of rs3128935 and rs9277972. The AUC was elevated to 0.619 by adding viral genotype in the prediction model. The results suggested that the genetic factors of host and pathogen were more important during resolution of virus.

### Interaction analysis

The interactions between the meaningful SNPs and potential risk factors were also analyzed (Table 5). For the protective effect of the rs3128935 C genotype, significant multiplicative interactions were found with high-risk population (*P*_interaction_ = 0.013) and age (*P*_interaction_ = 0.035). Compared to IDUs with the rs3128935 TT genotype, hemodialysis patients with the TT genotype had a decreased prevalence of persistent HCV infection (OR = 0.37, 95% CI = 0.20–0.69). Compared to subjects with the rs3128935 TT genotypes and ≥40 years old, subjects with the CT/CC genotypes but < 40 years old had a 70% decrease in risk of persistent HCV infection (OR = 0.30, 95% CI = 0.14–0.61).

**Table 5:** Interaction analysis between rs3128935 genotypes and high-risk population or age.

**a) rs3128935 genotypes and high-risk population**			
**Variables**	**Resolver N(%)**	**Chronic N(%)**	**OR (95% CI)**
TT genotypes with drug using	102 (41.5)	117 (53.7)	1.00
CT/CC genotypes with drug using	52 (21.1)	25 (11.5)	0.40 (0.24–0.74)
TT genotypes with HD	73 (29.7)	57 (26.1)	0.37 (0.20–0.69)
CT/CC genotypes with HD	19 (7.7)	19 (8.7)	0.48 (0.20–1.11)
*P* for multiplicative interaction			*P* = 0.013
**b) rs3128935 genotypes and age**			
**Variables**	**Resolver N(%)**	**Chronic N(%)**	**OR (95% CI)**
TT genotypes with ≥ 40 years	76 (30.8)	75 (34.4)	1.00
CT/CC genotypes with ≥ 40 years	22 (8.9)	24 (11.0)	1.09 (0.55–2.17)
TT genotypes with < 40 years	99 (40.2)	99 (45.4)	0.71 (0.41–1.25)
CT/CC genotypes with < 40 years	49 (20.0)	20 (9.2)	0.30 (0.14–0.61)
*P* for multiplicative interaction			*P* = 0.035

Logistic regression analyses adjusted for age, gender, high-risk population, and viral genotype.

Abbreviation: HD, hemodialysis.

## Discussion

A recent genome wide association study (GWAS) of the European and African populations revealed that there was a significant association between the rs4273729 marking HLA-DQB1*03:01 and spontaneous resolution of HCV infection [23]. Taken together with our preliminary work, we have the confidence that HLA regions also play a vital role on HCV infection outcomes in the Chinese population. In the present study, we investigated the role of antigen presentation-related genes on HCV susceptibility and clearance in subjects of IDUs and hemodialysis patients. Overall, 34 tagging-SNPs in 9 candidate genes (*HLA-DMA*, *HLA-DMB*, *HLA-DOA*, *HLA-DOB*, *TAP1*, *TAP2*, *LMP2*, *LMP7*, *tapasin*) were selected and analyzed.

The results indicated that *HLA-DOB* rs2071469 and *TAP2* rs1800454 were associated with susceptibility to HCV infection (Tables 2A and 4A). This association was still significant after FDR correction (S2 Table). The *TAP2* rs1800454 G > A mutation results in a missense mutation, and the *HLA-DOB* rs2071469 A > G mutation was located in the 5’UTR. Further functional studies are required to demonstrate the impact of these mutations on protein expression and activity. As shown in Table 2A, the significance for rs1800454 was greater in heterozygous than in homozygous subjects. It is reasonable to infer that the significance of homozygotes is expected to be stronger than heterozygotes. However, in our cohort, the sample size of homozygous subjects was too small. There were just 17 and 21 subjects carrying the AA genotype in the infected group and uninfected group, respectively. The significance of homozygosity might be underestimated in this case. A larger sample size may help reveal the real significance of the AA genotype. Despite the strong connection of these two SNPs with HCV susceptibility, high risk exposure and age were more valuable as predictors of infection outcomes.

The indispensible role of the human immune system was demonstrated during viral clearance. *HLA-DOA* rs3128935 and *tapasin* rs9277972 were independent factors of infection chronicity (Tables 2B and 4B). Factors other than genetic background had interaction with rs3128935 (Table 5). The risk of chronic infection was increased in IDUs and subjects ≥ 40 years old whose physiological condition was relatively weak. The results suggested that both innate immunity and physiological status were influential during viral clearance. We did not find any interactions between other factors which might be due to selection bias as well as other potential confounding factors. Viral genotype could increase the predictive value for chance of persistence. However, the area under the ROC curve was not ideal enough to make any convincing predictions, which indicated that the SNPs with stronger associations (for example, *IL28B*) or other factors should be considered for clinical applications. The *HLA-DOA* rs3128935 T > C mutation was located in the 3’UTR, which might modulate expression on either the transcriptional or translational levels. However, the *tapasin* rs9277972 A > T mutation was in an intron region. Further studies are warranted to elucidate the biological plausibility of these SNPs.

Another study in a European Caucasian population found that the G allele in exon 4 of *tapasin* was associated with outcomes of HCV infection, which not observed in our study [24]. The discrepancy among these studies may be due to the different study design as well as the participants with different genetic backgrounds. The inconsistencies among these studies also emphasized the benefits of data collection from a specific population in order to reveal unique disease-related effects within a given genetic background rather than in generalized population setting.

One weakness of this study is the relative small sample size. We calculated power in a dominant model. When combining all the subjects and comparing the infected with the uninfected, the power for rs1800454 was 0.812 and the power for rs2071469 was 0.887. When comparing the chronic patients with the resolvers, the power for rs9277972 and rs3128935 was 0.623 and 0.587, respectively. In this case, one possible explanation is the poor sample size (218 chronic patients and 246 resolvers). Both dialysis patients and IDUs were included for a larger sample size, although the two groups were not very comparable. Stratified analysis was performed, and unsurprisingly, the results were not consistent between the groups (S3 Table). The effect of rs2071469 was significant in IDUs but not in dialysis patients. The effect of the other three SNPs was significant in dialysis patients but not in IDUs.

Several other potential limitations need to be taken into consideration in this study. First, 34 SNPs were included in the present study, which may lead to potential false positive results due to multiple comparisons. Given that the FDR of rs3128935 and rs9277972 were 0.26, the relationship with HCV clearance was not significant after FDR-BH correction at 0.05 level. The results indicated that effect of most single SNP was weak and the disease was actually outcome of multiple factors. Second, selection bias may be induced during sample collection. Although only subjects with risk exposure of drug injecting/ hemodialysis were recruited, some uninfected controls may never have contact with the pathogen. The immune status of these subjects was compromised when compared with the healthy population, and therefore, there might be other unknown confounding factors involved in the analyses. Finally, little is known regarding the biological mechanism of the significant SNPs in the outcomes of HCV. Therefore, replication studies with functional characterizations of these SNPs are required to confirm our findings.

In conclusion, our study implicated that *HLA-DOA*, *HLA-DOB*, *TAP2*, and *tapasin* loci were candidate regions that had some novel marker SNPs for susceptibility to HCV infection and clearance of the virus in the Chinese population. Behavior exposure and physiological condition were also involved in disease development.

## Supporting Information

S1 Fig Prediction of HCV susceptibility. **(a) Importance blots of all variables in 1237 subjects.** (b) ROC curve for prediction of HCV susceptibility using the combination of rs2071469 and rs1800454. (c) ROC curve with the combination of rs2071469, rs1800454, and high-risk exposure (hemodialysis or injecting drug) to predict HCV susceptibility. (d) ROC curve with the combination of rs2071469, rs1800454, high-risk population, and age.
(TIF)

S2 Fig Prediction of HCV chronicity. (a) Importance blots of all variables in 464 HCV-positive subjects. (b) ROC curve for prediction of HCV chronicity using the combination of rs3128935 and rs9277972. (c) ROC curve with the combination of rs3128935, rs9277972, and viral genotype to predict HCV chronicity.
(TIF)

S1 Table Information of primers and probes for TaqMan allelic discrimination. Primers and probes of the 34 tagging-SNPs were designed for amplification.
(PDF)

S2 Table Comparison results of SNPs distribution in dominant, recessive, and additive models. All SNPs were searched for concrete location in genes. Each model was used to find the different distribution of SNPs in HCV resolvers versus HCV chronic cases and HCV chronic cases versus HCV resolvers.
(PDF)

S3 Table Stratified analysis in subgroups. Rs1800454, rs2071469, rs9277972 and rs3128935 used stratified analysis in dominant and additive models by population resource.
(PDF)
