# Supplementary figures and images for: Association of Polymorphisms in HLA Antigen Presentation-Related Genes with the Outcomes of HCV Infection
Source: PLoS One. 2015 Apr 13;10(4):e0123513. doi: 10.1371/journal.pone.0123513 (PMC4395248; doi:10.1371/journal.pone.0123513)

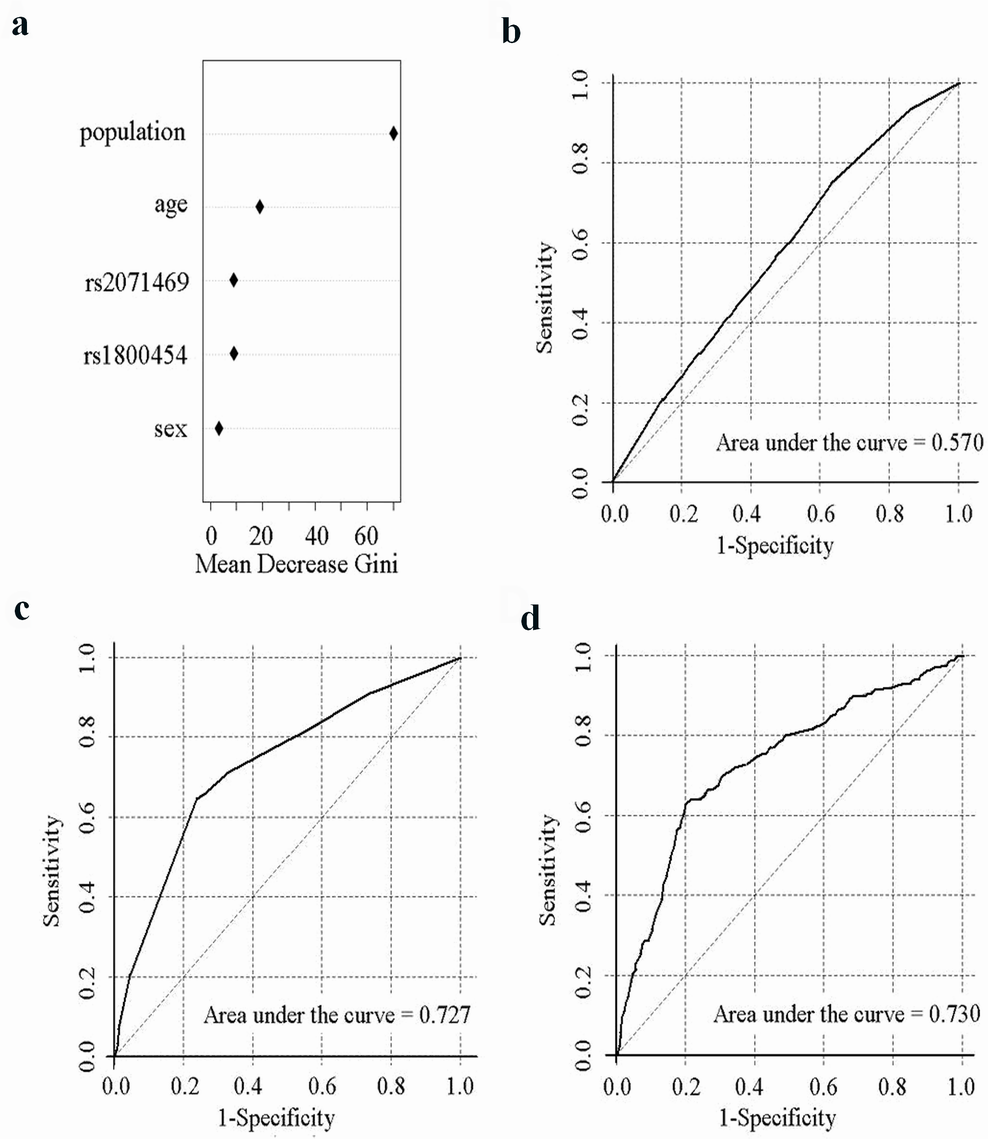

Supplement: S1 Fig — (a) Importance blots of all variables in 1237 subjects. (b) ROC curve for prediction of HCV susceptibility using the combination of rs2071469 and rs1800454. (c) ROC curve with the combination of rs2071469, rs1800454, and high-risk exposure (hemodialysis or injecting drug) to predict HCV susceptibility. (d) ROC curve with the combination of rs2071469, rs1800454, high-risk population, and age. (TIF) [file pone.0123513.s001.tif]

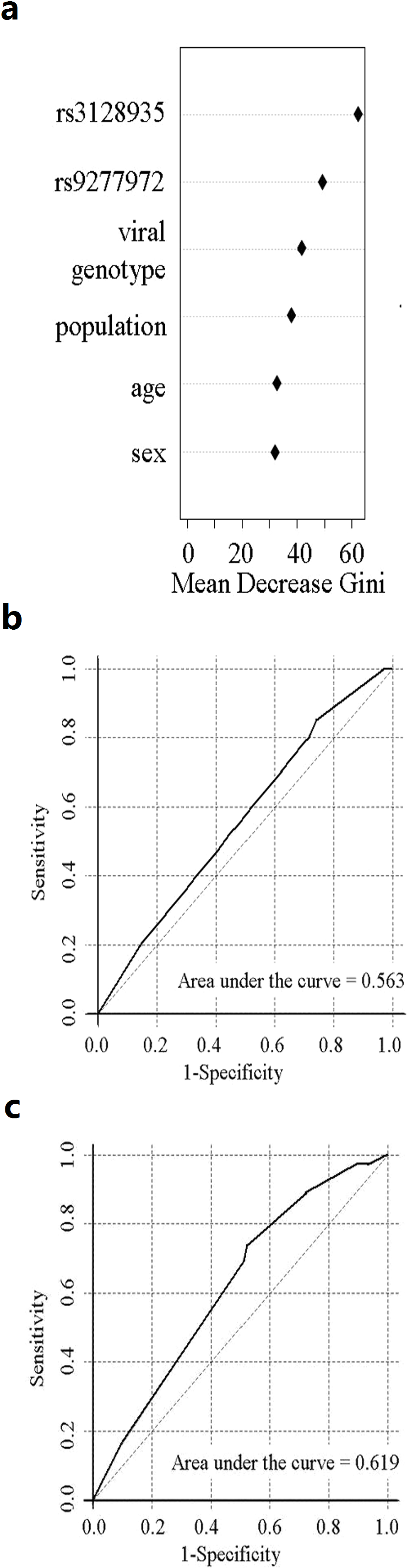

Supplement: S2 Fig — (a) Importance blots of all variables in 464 HCV-positive subjects. (b) ROC curve for prediction of HCV chronicity using the combination of rs3128935 and rs9277972. (c) ROC curve with the combination of rs3128935, rs9277972, and viral genotype to predict HCV chronicity. (TIF) [file pone.0123513.s002.tif]
