# Supplementary material for: Association of Polymorphisms in HLA Antigen Presentation-Related Genes with the Outcomes of HCV Infection
Source: PLoS One. 2015 Apr 13;10(4):e0123513. doi: 10.1371/journal.pone.0123513 (PMC4395248; doi:10.1371/journal.pone.0123513)
Supplement: S1 Table — Primers and probes of the 34 tagging-SNPs were designed for amplification. (PDF) [file pone.0123513.s003.pdf]

S1 Table. Information of primers and probes for TaqMan allelic discrimination

Polymorphism		Sequence(5'-3')
DMA rs1050391	Primer	F: AGGTCTTCTTCCAGGCAAGGA<br>R: GAGCTATAGACAGGAAGTGCTGAA
	Probe	FAM-CTAGACGTAGAAGCAG-MGB<br>HEX-AGCTAGACATAGAAGCAGA-MGB
DMA rs1063478	Primer	F: CCACCCATGCTGACAGTGA<br>R: AGTCCATCGACAGCTGAGACA
	Probe	FAM-ATCATTCCGTCCCTGTG-MGB<br>HEX-CATCATTCCATCCCTGT-MGB
DMA rs3135029	Primer	F: TCGCTTGGGTGTTCCTGTATAA<br>R: CAGGGCCCACGTGATCTG
	Probe	FAM-TAAGCTACAAATTCTGGCA-MGB<br>HEX-ATAAGCTACAAATTCGGGCAA-MGB
DMB rs23544	Primer	F: CCACTGTATTAGAGAGGACCTGGAA<br>R: ACCACCCACTCCCAAGAAAAT
	Probe	FAM-AGATGGATTCCCC-MGB<br>HEX-AGATGGGTTCCCC-MGB
DMB rs2071556	Primer	F: GTTGAAATCTAAGGAGGCTAGAATAGTGTA<br>R: GGAGCGTGCTTGCTTTGAA
	Probe	FAM-ATTGCCAAATTTACAGCC-MGB<br>HEX-TGCCAAATTTACATCCT-MGB
DOA rs408036	Primer	F: CCAGGCCTTGGCCAGTT<br>R: GTAACACACAATGGGCCAAATG
	Probe	FAM-TTGGCAGCCGTCCT-MGB<br>HEX-ATTGGCAGCCATC-MGB
DOA rs3128935	Primer	F: TGTCGGGTGGACATGTTCAC<br>R: GGATCCACATGGTCTGTGTTCTC
	Probe	FAM-AGAACACCGCTAACA-MGB<br>HEX-AGAACACCGCCAACA-MGB
DOA rs3129304	Primer	F: AAAACATACAAAGAGATAAATCACCATACC<br>R: TGAAAACCGTAATCTGTATTGCTCAT
	Probe	FAM-CATAGTTTATGTCAGGACC-MGB<br>HEX-CATAGTTTATGTCAAGACC-MGB
DOA rs376892	Primer	F: CTTGGCTGTGGTCTGGTAACTG<br>R: CCTTCCTAGTCCACCTCAGACCTT
	Probe	FAM-TAATCAGGTGCCATTGG-MGB<br>HEX-TAATCAGGTGCCATCGG-MGB
DOA rs369150	Primer	F: GAAAGAAAGGAACAGGGCATGAC<br>R: GGCGGGAAGGTCCAGAGA
	Probe	FAM-TGATGGGAACCTAGG-MGB<br>HEX-TGATGGGAGCCTAGG-MGB
DOA rs86567	Primer	F: GGTGCGGGTCTACAGATGGTT<br>R: GAGCAACAGTTATTGAGGAACTAGCAT

	Probe	FAM-TGGCCCCCCATTG-MGB
		HEX-TGGCCCACCATTG-MGB
DOA rs2284191	Primer	F: TCCTCCATCTCAGAGCATTATGAC
		R: TGTTGCTCAAACAACTTCATAGAGTTC
	Probe	FAM-CTTCCATAACTGTTGTCTAG-MGB
		HEX-TAACTGTTATCTAGTTTTCTGG-MGB
DOA rs6913008	Primer	F: GTCCTGTTCAGAGTCATCCACTTT
		R: TCCTCATCATCATGGGCACAT
	Probe	FAM-CCCAGACTCCCGG-MGB
		HEX-CCCAGACTCCTGG-MGB
DOA rs2582	Primer	F: TGATCCTTCTGAGAGAAATGACTTGT
		R: CACAGCGGGATGCACTTAAA
	Probe	FAM-TGTGACAGACCCTGC-MGB
		HEX-TGTGACAGCCCCTG-MGB
DOA rs416622	Primer	F: CAGCCTGGTGACAGAGTGAGA
		R: TCACCCAGACCTACTGAATTAGAATCT
	Probe	FAM-AGACAGCCCCCCTGT-MGB
		HEX-AGACAGCCTCCCTGTT-MGB
DOA rs453779	Primer	F: GTCACCCGTGGAGGCACTA
		R: AACGTCCCTTAATCCCAGTCCTA
	Probe	FAM-AGGAACAGGCCCTG-MGB
		HEX-AGGAACGGGCCCTG-MGB
DOA rs1044429	Primer	F: TCACACAAAGAGGGTTTCTGTTACTG
		R: GAATAAGTTGAAATCAATGACCAGAAGA
	Probe	FAM-TGAGATGATTCTCCTCCAC-MGB
		HEX-TGAGATGATTTTCCTCCAC-MGB
DOB rs2857111	Primer	F: TCTCTTGCCTCCGTTCTCATTC
		R: TGCTACATATTTCTAAAAGCCACTCTCATA
	Probe	FAM-TCCCCTCCCTGGAGA-MGB
		HEX-CTCCCCTCCCTAGAG-MGB
DOB rs11244	Primer	F: CAAGGGCATTGTCCAGAAACTC
		R: GCTGGACCACAGAAAAGTAAATGA
	Probe	FAM-ACTCCTTCCAGTCCCA-MGB
		HEX-CTCCTTCCAGCCCCA-MGB
DOB rs1383258	Primer	F: TTACCAGACACGTTTAGAATGGATTC
		R: GAGTTCACAGCACATTGTAATTATTGG
	Probe	FAM-AGAAGAGATGAGAGAGTC-MGB
		HEX-CAAGAGAAGAGACGAGAG-MGB
DOB rs2071469	Primer	F: GAAGCCAGAAAAACAGCGATTTAT
		R: AGGGGACCCACCCAGAAC
	Probe	FAM-TGAATTTTATGAACTCATTC-MGB
		HEX-TGAATTTTATGAGCTCATTC-MGB
DOB rs2071472	Primer	F: GACTGGATTCCTCCATGACTCAA
		R: CATGCCAATTCTTGCATACACA

	Probe	FAM-AACAGAGCAATTGTT-MGB
		HEX-AACAGAGCAATTATT-MGB
DOB rs7383287	Primer	F: CGTAATTTACCAGGCATGGGTTT
		R: CAGTCAGCCTTTGCCTGAATC
	Probe	FAM-TTCCAGAAGATTTTG-MGB
		HEX-TTTCCAGAAGACTTTG-MGB
DOB rs2856997	Primer	F: CCAAATCCAATGCTAGCTAGAGAAA
		R: ATGGGCTGTGAGAATCTGTAACC
	Probe	FAM-CATGGAGTTACCCCC-MGB
		HEX-CCATGGAGTTACCACC-MGB
DOB rs2071471	Primer	F: CATGCCAATTCTTGCATACACACT
		R: GGTGGCTCTGCTAGTGAATCTGA
	Probe	FAM-CTGGAGAATCTGTGC-MGB
		HEX-CTGGAGAGTCTGTGC-MGB
DOB rs2071475	Primer	F: GGTCCTCTCTGGGTACACTGTCA
		R: GGTTTTCTTTCACGGTGTCTCAT
	Probe	FAM-CTAGGAAGGGAGGAAA-MGB
		HEX-ACTAGGAAGAGAGGAAA-MGB
DOB rs2856995	Primer	F: CTCCCCTCCCTAGAGAATCCA
		R: AAGAAACAGAATGGGATTCAACAGA
	Probe	FAM-TGTCTGGTATGTCTCTAA-MGB
		HEX-TGTCTGGTAAGTCTC-MGB
TAP1 rs1135216	Primer	F: CACACATGTGGCTATACCGTTCTC
		R: TCGCTGACCCCCTGACA
	Probe	FAM-TGCAGAGGTAGGCG-MGB
		HEX-TCTGCAGAGGTAGACG-MGB
TAP2 rs1800454	Primer	F: CCTGGAACGCGCCTTGTA
		R: CCTTTCACAACCACTCTGGTATCTT
	Probe	FAM-TGCTCGTAAGGAGG-MGB
		HEX-CTGCTCATAAGGAGG-MGB
LMP2 rs17587	Primer	F: TGAACCGAGTGTTTGACAAGCT
		R: GCTGAACCAGAGAGTGCACAGT
	Probe	FAM- CTGCACGAGCGCA-MGB
		HEX- CTGCACGAGCACAT-MGB
LMP7 rs2071543	Primer	F: GCGACCCTCCACTCCTCA
		R: GGACACTACAGTTTCTCTATGCGATCT
	Probe	FAM- CCGACCTTCATTCC-MGB
		HEX- CCGACCTGCATTC-MGB
tapasin rs9277972	Primer	F: GTCTAGGTCCTTCAGGTAGAAGTAATCTTT
		R: CTAAGTGAAATTGCATACTGTTTTTACTCTAC
	Probe	FAM-CCTATAAGGTTAAACTGTTCT-MGB
		HEX-CCTATAAGGTTTAACTGTTCT-MGB
tapasin rs1059288	Primer	F: TGGGCCTTAGGTCCCTATGC
		R: AAGTGATCGTGTGAGTCGTCGTT

tapasin rs2282851	Probe	FAM-CAGACAGGCCGGTC-MGB<br>HEX-ACAGACAGGCCAGTC-MGB
	Primer	F: CCTCATTCTTGAATTATCTGCACAGT<br>R: GCCCAGGAGTCAGAAGCTTTT
	Probe	FAM-CCACGTCTCAGCCTA-MGB<br>HEX-CCACGTCCCAGCCT-MGB
