# Supplementary material for: Association of Polymorphisms in HLA Antigen Presentation-Related Genes with the Outcomes of HCV Infection
Source: PLoS One. 2015 Apr 13;10(4):e0123513. doi: 10.1371/journal.pone.0123513 (PMC4395248; doi:10.1371/journal.pone.0123513)
Supplement: S2 Table — All SNPs were searched for concrete location in genes. Each model was used to find the different distribution of SNPs in HCV resolvers versus HCV chronic cases and HCV chronic cases versus HCV resolvers. (PDF) [file pone.0123513.s004.pdf]

S2 Table. Comparison results of SNPs distribution in dominant, recessive, and additive models

SNPs	Location	Dominant [a]		Recessive [a]		Additive [a]		Dominant [b]		Recessive [b]		Additive [b]	
		***P* Value**	**FDR***	***P* Value**	**FDR***	***P* Value**	**FDR***	***P* Value**	**FDR***	***P* Value**	**FDR***	***P* Value**	**FDR***
DMA rs1050391	intron(C>T)	8.52×10^{-1}	0.98	2.95×10^{-1}	0.80	6.09×10^{-1}	0.81	2.56×10^{-1}	0.92	1.56×10^{-1}	0.77	6.66×10^{-1}	0.99
DMA rs1063478	Missense(C>T)	4.58×10^{-1}	0.77	8.15×10^{-2}	0.80	9.01×10^{-1}	0.96	5.33×10^{-1}	0.92	3.56×10^{-1}	0.77	8.64×10^{-1}	0.99
DMA rs3135029	5'near gene(C>A)	8.64×10^{-1}	0.98	1.33×10^{-1}	0.80	5.16×10^{-1}	0.81	2.49×10^{-1}	0.92	1.06×10^{-1}	0.77	7.26×10^{-1}	0.99
DMB rs23544	intron(G>A)	9.83×10^{-1}	0.98	7.03×10^{-1}	0.92	8.42×10^{-1}	0.95	7.19×10^{-1}	0.93	7.25×10^{-1}	0.93	6.64×10^{-1}	0.99
DMB rs2071556	intron(C>A)	8.03×10^{-1}	0.98	8.01×10^{-1}	0.92	9.60×10^{-1}	0.99	4.65×10^{-1}	0.92	4.26×10^{-1}	0.77	3.57×10^{-1}	0.94
DOA rs408036	3'UTR(G>A)	3.71×10^{-1}	0.66	1.97×10^{-1}	0.80	1.91×10^{-1}	0.42	3.26×10^{-1}	0.92	1.97×10^{-1}	0.77	9.87×10^{-1}	0.99
DOA rs3128935	3'UTR(T>C)	5.95×10^{-2}	0.34	5.54×10^{-1}	0.92	6.52×10^{-2}	0.42	3.14×10^{-2}	0.53	6.20×10^{-2}	0.77	1.17×10^{-2}	0.26
DOA rs3129304	3'UTR(A>G)	6.77×10^{-1}	0.89	4.70×10^{-1}	0.92	8.32×10^{-1}	0.95	6.37×10^{-1}	0.93	9.32×10^{-1}	0.98	6.43×10^{-1}	0.99
DOA rs376892	3'UTR(C>T)	3.25×10^{-1}	0.65	1.28×10^{-1}	0.80	1.61×10^{-1}	0.42	7.65×10^{-1}	0.93	7.51×10^{-1}	0.93	7.13×10^{-1}	0.99
DOA rs369150	intron(G>A)	6.26×10^{-1}	0.89	8.15×10^{-1}	0.92	6.43×10^{-1}	0.81	8.20×10^{-1}	0.93	2.25×10^{-1}	0.77	6.54×10^{-1}	0.94
DOA rs86567	intron(A>C)	7.35×10^{-1}	0.93	4.68×10^{-1}	0.92	8.59×10^{-1}	0.95	7.19×10^{-1}	0.93	2.76×10^{-1}	0.77	7.10×10^{-1}	0.94
DOA rs2284191	intron(G>A)	9.66×10^{-1}	0.98	1.66×10^{-1}	0.80	6.29×10^{-1}	0.81	5.24×10^{-1}	0.92	9.79×10^{-1}	0.98	8.95×10^{-1}	0.99
DOA rs6913008	intron(C>T)	1.05×10^{-1}	0.34	6.50×10^{-1}	0.92	1.33×10^{-1}	0.42	8.99×10^{-1}	0.93	3.50×10^{-1}	0.77	6.50×10^{-1}	0.94
DOA rs2582	3'UTR(C>A)	5.58×10^{-2}	0.34	9.20×10^{-1}	0.95	1.05×10^{-1}	0.42	6.58×10^{-1}	0.93	5.93×10^{-1}	0.88	8.71×10^{-1}	0.99
DOA rs416622	3'UTR(G>A)	7.88×10^{-2}	0.34	6.42×10^{-1}	0.92	1.29×10^{-1}	0.42	7.96×10^{-1}	0.93	7.42×10^{-1}	0.93	9.83×10^{-1}	0.99
DOA rs453779	intron(C>T)	8.26×10^{-2}	0.34	5.25×10^{-1}	0.92	1.15×10^{-1}	0.42	5.98×10^{-1}	0.92	8.99×10^{-1}	0.98	7.41×10^{-1}	0.99
DOA rs1044429	3'UTR(G>A)	8.91×10^{-2}	0.34	7.36×10^{-1}	0.92	6.39×10^{-1}	0.81	9.03×10^{-1}	0.93	2.08×10^{-1}	0.77	2.24×10^{-1}	0.99
DOB rs2857111	intron(A>G)	6.52×10^{-1}	0.89	7.62×10^{-1}	0.92	6.26×10^{-1}	0.81	2.48×10^{-1}	0.92	6.16×10^{-1}	0.88	2.45×10^{-1}	0.99
DOB rs11244	3'UTR(C>T)	1.03×10^{-1}	0.34	3.35×10^{-1}	0.80	8.43×10^{-2}	0.42	9.33×10^{-1}	0.93	3.10×10^{-1}	0.77	7.64×10^{-1}	0.99
DOB rs1383258	intron(G>A)	2.76×10^{-1}	0.63	1.08×10^{-1}	0.80	6.01×10^{-1}	0.81	5.27×10^{-1}	0.92	9.80×10^{-1}	0.98	4.57×10^{-1}	0.99
DOB rs2071469	5'UTR(A>G)	7.42×10^{-4}	0.03	3.43×10^{-4}	0.01	5.19×10^{-5}	0.00	9.17×10^{-1}	0.93	4.14×10^{-1}	0.77	7.88×10^{-1}	0.99
DOB rs2071472	intron(G>A)	5.27×10^{-1}	0.78	3.44×10^{-1}	0.80	3.49×10^{-1}	0.66	2.38×10^{-1}	0.92	6.09×10^{-1}	0.88	5.69×10^{-1}	0.94

DOB rs7383287	synonymous(A>G)	4.73×10^{-1}	0.77	9.69×10^{-1}	0.97	2.91×10^{-1}	0.58	3.97×10^{-1}	0.92	---[#]	---[#]	3.97×10^{-1}	0.94
DOB rs2856997	intron(T>G)	1.11×10^{-1}	0.34	3.07×10^{-1}	0.80	1.06×10^{-1}	0.42	8.02×10^{-1}	0.93	1.60×10^{-1}	0.77	4.98×10^{-1}	0.94
DOB rs2071471	synonymous(C>T)	5.04×10^{-1}	0.78	8.41×10^{-1}	0.92	5.73×10^{-1}	0.81	5.12×10^{-1}	0.92	5.06×10^{-1}	0.84	6.71×10^{-1}	0.99
DOB rs2071475	intron(C>T)	2.43×10^{-1}	0.59	2.05×10^{-1}	0.80	1.43×10^{-1}	0.42	3.14×10^{-1}	0.92	8.55×10^{-1}	0.97	4.64×10^{-1}	0.94
DOB rs2856995	intron(T>A)	1.11×10^{-1}	0.34	7.39×10^{-1}	0.92	1.93×10^{-1}	0.42	5.39×10^{-1}	0.92	6.94×10^{-2}	0.77	1.68×10^{-1}	0.94
TAP1 rs1135216	Missense(A>G)	9.40×10^{-1}	0.98	8.33×10^{-1}	0.92	9.96×10^{-1}	1.00	3.41×10^{-1}	0.92	4.42×10^{-1}	0.77	2.84×10^{-1}	0.99
TAP2 rs1800454	Missense(G>A)	4.62×10^{-3}	0.08	3.52×10^{-1}	0.80	6.79×10^{-3}	0.12	2.49×10^{-1}	0.92	3.23×10^{-1}	0.77	5.29×10^{-1}	0.94
LMP2 rs17587	Missense(G>A)	1.40×10^{-1}	0.40	3.38×10^{-1}	0.80	1.12×10^{-1}	0.42	4.03×10^{-1}	0.92	7.82×10^{-1}	0.93	4.21×10^{-1}	0.94
LMP7 rs2071543	Missense(C>A)	3.70×10^{-1}	0.66	9.13×10^{-1}	0.95	4.18×10^{-1}	0.75	1.79×10^{-1}	0.92	1.21×10^{-1}	0.77	9.30×10^{-2}	0.94
tapasin rs9277972	intron(A>T)	1.65×10^{-1}	0.43	7.05×10^{-1}	0.92	1.74×10^{-1}	0.42	2.36×10^{-2}	0.53	3.44×10^{-1}	0.77	2.16×10^{-2}	0.26
tapasin rs1059288	5'near gene(T>C)	9.27×10^{-1}	0.98	6.49×10^{-1}	0.92	8.65×10^{-1}	0.95	2.82×10^{-1}	0.92	4.16×10^{-1}	0.77	2.41×10^{-1}	0.94
tapasin rs2282851	intron(C>T)	2.97×10^{-1}	0.63	2.59×10^{-1}	0.80	1.98×10^{-1}	0.42	5.66×10^{-1}	0.92	7.88×10^{-1}	0.93	5.67×10^{-1}	0.99

[a] HCV infected cases (including both resolvers and chronic cases) versus uninfected subjects; Logistic regression analyses adjusted for age, gender, and high-risk population.

[b] HCV chronic cases versus HCV resolvers; Logistic regression analyses adjusted for age, gender, high-risk population, and viral genotype.

* FDR, false discovery rate; Multiple testing: using FDR.

[#] For rs7383287, GG homozygote was rarely seen in HCV-infected subjects, therefore the recessive model was not able to be used here.
