# Supplementary material for: Association of Polymorphisms in HLA Antigen Presentation-Related Genes with the Outcomes of HCV Infection
Source: PLoS One. 2015 Apr 13;10(4):e0123513. doi: 10.1371/journal.pone.0123513 (PMC4395248; doi:10.1371/journal.pone.0123513)
Supplement: S3 Table — Rs1800454, rs2071469, rs9277972 and rs3128935 used stratified analysis in dominant and additive models by population resource. (PDF) [file pone.0123513.s005.pdf]

S3 Table. Stratified analysis in subgroups

		Uninfected (N)	Infected (N)	OR (95% CI)	*P* value
rs1800454					
GG/AG/AA	HD patients	445/130/15	116/45/7		
	IDUs	149/28/6	206/80/10		
Dominant	HD patients			1.38(0.94-2.01)	0.097
	IDUs			1.91(1.22-2.99)	0.004
Additive	HD patients			1.33(0.98-1.83)	0.076
	IDUs			1.59(1.09-2.33)	0.016
rs2071469					
AA/AG/GG	HD patients	280/195/115	62/72/34		
	IDUs	149/33/1	238/57/1		
Dominant	HD patients			1.21(0.97-1.51)	0.091
	IDUs			1.07(0.67-1.71)	0.784
Additive	HD patients			1.54(1.09-2.18)	0.014
	IDUs			1.05(0.67-1.65)	0.837
		**Resolver (N)**	**Chronic (N)**	**OR (95% CI)**	***P* value**
rs9277972					
AA/AT/TT	HD patients	74/17/1	55/19/2		
	IDUs	132/21/1	109/31/2		
Dominant	HD patients			1.47(0.71-3.03)	0.299
	IDUs			1.82(1.00-3.30)	0.049
Additive	HD patients			1.40(0.72-2.75)	0.322
	IDUs			1.74(1.02-3.04)	0.047
rs3128935					
TT/CT/CC	HD patients	73/17/2	57/18/1		
	IDUs	102/46/6	117/25/0		
Dominant	HD patients			1.28(0.62-2.64)	0.503
	IDUs			0.42(0.24-0.73)	0.002
Additive	HD patients			1.17(0.61-2.23)	0.632
	IDUs			0.41(0.25-0.68)	0.001

HD, hemodialysis; IDUs, injecting drug users.
